# Therapeutic strategies for multi-resistant *Escherichia coli* in urinary tract infections—a cause for concern

**DOI:** 10.1093/jacamr/dlag081

**Published:** 2026-05-21

**Authors:** Rekha Unni, Ruby Varghese, Benita Arakal, Lochana Padmanabhan, Srijita Karmakar, Achinthya Sampathila, Yogesh Bharat Dalvi, Paul Livingstone

**Affiliations:** Post Graduate and Research Department of Chemistry, Christian College, University of Kerala, Chengannur, Kerala 689122, India; Department of Chemistry and Biochemistry, School of Sciences, Jain Deemed to be University, Bengaluru, Karnataka 560027, India; School of Sports and Health Sciences, Cardiff Metropolitan University, Llandaff Campus, Cardiff CF5 2YB, UK; Department of Chemistry and Biochemistry, School of Sciences, Jain Deemed to be University, Bengaluru, Karnataka 560027, India; Department of Chemistry and Biochemistry, School of Sciences, Jain Deemed to be University, Bengaluru, Karnataka 560027, India; Department of Chemistry and Biochemistry, School of Sciences, Jain Deemed to be University, Bengaluru, Karnataka 560027, India; Pushpagiri Institute of Medical Sciences, Pushpagiri Research Center, Thiruvalla, Kerala 689101, India; School of Sports and Health Sciences, Cardiff Metropolitan University, Llandaff Campus, Cardiff CF5 2YB, UK

## Abstract

Antimicrobial resistance (AMR) is a serious and growing public health concern driven by multiple factors. Urinary tract infection (UTI) is one of the infections commonly associated with AMR, and more than 80% of UTIs are caused by *Escherichia coli*. MDR *E. coli* is a significant problem that requires urgent intervention, particularly in complicated UTIs (cUTIs) with underlying comorbidities. Although prevalence rates of MDR *E. coli* vary globally, it remains a critical clinical and economic challenge on every continent. The majority of MDR *E. coli* strains produce ESBLs, followed by AmpC enzymes, making them generally resistant to penicillins, cephalosporins and aztreonam. While there are some therapeutic options for uncomplicated UTIs (uUTIs), choices are limited for cUTIs, with carbapenems being the mainstay of treatment. However, carbapenem resistance is on the rise, severely impeding clinical regimens. With very few options available, combinations of antimicrobial agents for synergistic activity are increasingly relied upon for carbapenem-resistant *E. coli*. This situation has compelled the search for novel antimicrobial agents, which are slowly entering the market, although not as quickly as AMR is spreading worldwide. Current focus is shifting towards alternative antimicrobial therapies that exhibit broad-spectrum bactericidal activity and immunomodulatory effects, considered for both preventive and therapeutic strategies. A multi-disciplinary approach combining novel antimicrobials, innovative therapies and robust infection control measures will be essential to effectively manage MDR *E. coli* in uUTIs and cUTIs and mitigate the global threat of antibiotic resistance.

## Introduction

Urinary tract infections (UTIs) are among the most common infectious diseases, leading to millions of visits to clinics and hospitalizations annually. They are more prevalent in women, with a lifetime risk of over 60%. UTIs contribute to 10%–30% of sepsis cases, with complicated UTIs (cUTIs) being the leading cause of urosepsis in older adults. Early diagnosis and treatment with antibiotics and source control are crucial. *Escherichia coli* is the primary pathogen, followed by *Enterobacter*, *Proteus*, *Pseudomonas aeruginosa* and *Klebsiella*. Of these, *E. coli* causes more than 80% of all UTIs. The rise of MDR in *E. coli* worsens patient outcomes, increases treatment costs, and poses a global health threat. MDR infections, once hospital-associated, are now common in the community, complicating management. Addressing antimicrobial resistance is essential to improving patient care and reducing the burden of recurrent and hospital-acquired UTIs.^1^

UTIs are classified into uncomplicated and complicated, based on the presence of underlying risk factors and comorbidities. Uncomplicated UTIs (uUTIs) are those that occur in men and women without fever, and the infection is confined to the bladder.^2^ These infections, again, are mostly caused by *E. coli* and present with symptoms like dysuria, frequency, urgency and suprapubic pain. They usually respond well to short courses of antibiotics. In contrast, cUTIs occur in individuals presenting with one or more of the following: fever, pyelonephritis, indwelling urinary catheters, prostatitis or bacteraemia.^2^ These infections involve a broader spectrum of pathogens, with more than 50% of them caused by *E. coli* and require more aggressive treatment with prolonged antibiotic therapy.^2,3^ When a complicated UTI is not promptly treated, it can progress to urosepsis, a life-threatening organ dysfunction caused by a dysregulated host response to infection.^4^ The pathophysiology of urosepsis involves bacterial ascent from the lower urinary tract to the kidneys, leading to pyelonephritis, followed by bacteraemia and systemic inflammatory response syndrome. If not addressed promptly, this can advance to septic shock, a subset of sepsis in which underlying circulatory and cellular/metabolic abnormalities are profound enough to substantially increase mortality.^4^ Patients with indwelling catheters, diabetes and immunosuppression are at a higher risk of developing urosepsis, which has a high morbidity and mortality rate. The growing prevalence of MDR Gram-negative bacteria adds up to the issue, significantly impacting the treatment of complicated UTIs and urosepsis. MDR pathogens such as ESBL producing and carbapenem-resistant *E. coli* (CREC) exhibit resistance to multiple antibiotic classes, making infections more difficult to treat. These bacteria utilize mechanisms like beta-lactamase production, efflux pumps, porin mutations and biofilm formation to evade antibiotic effects. The increasing resistance rates lead to treatment failures, prolonged hospital stays and higher mortality in urosepsis patients.^5,6^

Epidemiologically, complicated UTIs account for 30%–50% of all UTIs in hospitalized patients, while urosepsis contributes to 20%–30% of all sepsis cases, with mortality rates ranging from 20% to 40% depending on the presence of MDR infections. Catheter-associated UTIs (CAUTI) are a major contributor to healthcare-associated infections, and the prevalence of MDR pathogens is steadily rising. In some countries, ESBL-producing *E. coli* account for 40%–50% of complicated UTIs, while CREC infections are observed in 5%–10% of severe cases.^6^ The management of complicated UTIs and urosepsis involves both empirical and targeted antibiotic therapy. Empirical therapy is initiated before pathogen identification, using broad-spectrum antibiotics such as piperacillin–tazobactam, cefepime, carbapenems, aminoglycosides or colistin based on various epidemiological factors. However, due to the risk of antibiotic resistance, a targeted approach is preferred once culture and susceptibility results are available, allowing for de-escalation to more specific antibiotics. For example, ESBL infections may require carbapenems, while CREC infections may need newer agents like ceftazidime–avibactam or meropenem–vaborbactam.^6^

The increasing incidence of antimicrobial resistance necessitates the development of alternative antimicrobial agents. Novel β-lactam/β-lactamase inhibitors like ceftazidime–avibactam, meropenem–vaborbactam and imipenem–cilastatin–relebactam offer promising treatment options against resistant pathogens. Additionally, non-traditional antimicrobials such as plazomicin and eravacycline are being explored for MDR uropathogens. Bacteriophage therapy, antimicrobial peptides (AMPs), monoclonal antibodies targeting bacterial virulence factors and vaccine development against uropathogenic *E. coli* and are also under investigation as future treatment strategies. Given the rising global threat of MDR infections, judicious antibiotic use, enhanced infection control measures and the development of novel therapeutic approaches are essential to improve outcomes in complicated UTIs and urosepsis.^7^

## Epidemiology of MDR *E. coli*

Epidemiology of MDR *E. coli* is largely relied on geographic variability, healthcare infrastructure and socio-economic disparities. At a continental level, the evolving trends of MDR *E. coli* although varied between countries, clearly is on the rise globally.^8^

### Asia

Asia is a major hotspot for MDR *E. coli* due to widespread antibiotic use and limited regulation.^9^ Across multiple regions within the Asian continent, females steadily exhibit a higher prevalence of UTIs in contrast to males. For instance, a cross-sectional urine culture study in Pakistan using a non-probability purposive sampling technique reported that 70% of the 313 affected individuals were females.^10^ This aligns with findings from a multi-centre, hospital-based cross-sectional study in Iran, which utilized convenience sampling of *E. coli* isolates obtained from routine urine cultures of UTI patients.^11^ In that study, females and children under 10 years accounted for a significant proportion (73% of 110 *E. coli* isolates) of UTI cases. However, with MDR uropathogens, single-centre, cross-sectional studies that used consecutive sampling with urine samples collected from 4500 patients from Bangladesh suspected with community-acquired UTI reported a 73% higher prevalence among male patients.^12^  *E. coli* resistance to penicillins and cephalosporins was a recurrent theme across several reports in Asia.^9^ For instance, 80% resistance to amoxicillin, 63% to nalidixic acid, 58% to tetracycline and 54% to cefepime was observed in the analysis of 132 *E. coli* isolates from a single-centre, cross-sectional laboratory-based study using convenience sampling of urine samples from UTI patients in Jordan.^13^ This parallels findings from a hospital-based study in Pakistan using consecutive sampling, where 44% out of 100 uropathogenic *E. coli* isolates were MDR, exhibiting resistance to ampicillin, cefixime and ceftriaxone.^14^ Carbapenem resistance in Saudi Arabia, was linked to NDM1 and OXA-181 genes.^15^ A laboratory-based cross-sectional study utilizing genomic characterization on eight MDR *E. coli* strains purposively selected from urines of hospital-acquired UTI Sri Lankan patients harboured multiple carbapenemase, AmpC and ESBL genes.^16^ Similarly, a hospital-based cross-sectional study from Northern India reported the predominance of blaCTX-M-15 and carbapenemase genes among 343 drug-resistant *E. coli* from a consecutive sampling of 1516 urine samples presented with bacteriuria.^17^ Alarmingly, an estimated prevalence of mcr-1 plasmid-mediated colistin resistance (last-resort antibiotic) was 11.5% in Asia.^18^

### Africa

Limited surveillance infrastructure in many African countries hampers accurate estimation of MDR *E. coli* prevalence.^19^ A retrospective study of urine cultures from paediatric patients suspected of UTI conducted in Abuja, Nigeria, on paediatric patients with suspected UTIs identified 29% *E. coli* as the primary causative agent from 543 urine cultures, with amoxicillin–clavulanate and cotrimoxazole presenting multiple antimicrobial resistance index scores of 0.36 and 0.38, respectively, demonstrating poor efficacy.^20^ Similarly, an observational retrospective record-based study on a consecutive sampling of 469 UTI patients in Alexandria, Egypt, found that MDR *E. coli* to be the most frequently isolated bacterium in both community-acquired (68%) and hospital-acquired UTIs (49%).^21^ Furthermore, high resistance to cephalosporins (90%), trimethoprim–sulfamethoxazole (TMP–SMX) (63%) and ciprofloxacin (53%), were reported among those 469 UTI patients. Geographic differences in antibiotic resistance patterns were also evident in East Africa.^22^ A hospital-based study across Kenya, Tanzania and Uganda reported *E. coli* as the predominant UTI pathogen, with Uganda having the highest proportions of MDR (52%) among 2653 patients with UTI. Notably, *E. coli* from Kenya exhibited lower resistance rates to ampicillin (65%), amoxicillin/clavulanic acid (23%), trimethoprim (74%), ciprofloxacin (46%), ceftriaxone (29%) and ceftazidime (23%) compared with isolates from Tanzania and Uganda among 2653 patients with UTI.^22^ Hospital-based studies from Cameroon, reported 77% of 144 *Enterobacterales* were ESBL-producing strains and 6% resistance to imipenem.^23^

### Europe and the UK

MDR *E. coli* is an escalating concern in UTIs across Europe and the UK, with substantial resistance observed in community and hospital environments.^24,25^ Surveillance programmes such as the European Antimicrobial Resistance Surveillance Network indicate a rising prevalence of ESBL-producing *E. coli*, particularly in southern and eastern countries.^26,27^ The 2024 report states that the MDR *E. coli* rates were 11.03 per 100 000 population, which is 5.9% higher than the baseline of the 2019 report, in bloodstream infections. Although this is not significant, there is definitely a rising trend that reinstates the antibiotic stewardship initiatives to be strengthened in Europe and the UK. A cross-sectional study based on 775 *E. coli* isolates out of a consecutive sampling of 1280 clinical urine samples from six European countries namely Sweden, Germany, Latvia, Poland, Russia and Finland revealed that patients face a heightened risk of uncomplicated UTIs, which are caused by MDR *E. coli*, although resistance rates to nitrofurantoin, fosfomycin and mecillinam remain below 10%.^27^ Nevertheless, trimethoprim and TMP–SMX, are still recommended as first-line treatments despite notable resistance. In the UK, a hospital-based study on 199 *E. coli* isolates causing UTIs from the Norfolk region revealed that 7% of strains were classified as MDR, with significant resistance observed to ampicillin (52%) and trimethoprim (36%) in hospital-acquired infections.^28^ Also, nitrofurantoin resistance has been associated with MDR *E. coli* in many instances in the UK.^29^ Similar concerns arise where *E. coli* accounted for 65% of tertiary-care hospital infections out of a total of 354 patients with UTIs as per the results from a retrospective hospital-based study in Eastern Europe. Among these isolates, there were 17 extensively drug-resistant (XDR), 11 pan-drug-resistant and 126 MDR, including CREC and ESBL producers.^30^ In southern Europe, a hospital-based study in Crete, Greece, reported 16% MDR or ESBL-producing *E. coli* out of a consecutive sampling of 204 UTI samples. Paediatric populations are also affected; a hospital-based study from southern Greece found that 77% *E. coli* isolates from a consecutive sampling of 218 children hospitalized with community-acquired UTIs exhibited high resistance to ampicillin (42%), piperacillin (40%) and amoxicillin–clavulanic acid (29%), with 13% classified as MDR and 4% producing ESBLs.^31,32^

### Oceania

In oceanic countries like Australia, antimicrobial resistance among UPEC isolates from hospitalized patients disclosed an unhinging pattern. In Australia, resistance rates in UTIs are low (9.7%) with *E. coli* ST131 the predominant MD lineage isolated.^33^ Similarly, 21 UPEC ST131 isolates from UTI patients from two large hospitals in Brisbane, Australia, included a MDR strain, *E. coli* EC958 of the globally disseminated ST131 lineage, according to a molecular-based retrospective study. This isolate produced CTX-M-15 ESBL and CMY-23 AmpC cephalosporinases and exhibited resistance to ciprofloxacin, while also harbouring key virulence genes associated with UPEC.^34^ Community-onset extended-spectrum cephalosporin-resistant *E. coli* remains relatively uncommon in Australia and New Zealand and emerging evidence insinuates that international travel and prior antibiotic-use interject a gradual increase in resistance.^35^ Interestingly, MDR *E. coli* is seen in higher proportions in males compared with females in Australia, attributed to prior treatment UTIs in males.^36^ Within the Pacific region, a retrospective analysis from Fiji documented a marked rise in ESBL-producing *E. coli* isolates, with an increased prevalence of 34% from a sampling of 3743 urine samples of patients from Colonial War Memorial Hospital in 2022. Alarmingly, this retrospective study highlighted the emergence of CREC, as susceptibility to meropenem declined significantly from 99% to 79% in 2022.^37^ Consistent with this trend, a systematic review of antimicrobial resistance in Pacific Island countries between 2011 and 2017 reported an 85% resistance rate to penicillins, while fluoroquinolone and cephalosporin resistance remained low at 12%.^38^

### North America

A significant burden of MDR *E. coli* infections causing UTIs is a growing concern across the USA and Mexico. In the USA, as per a hospital-based study, 233 974 isolates of *E. coli* from a consecutive sampling of 174 185 individuals who had greater than or equivalent to one UPEC uUTI at Kaiser Permanente Southern California exhibited a 29% significant resistance to penicillins, fluoroquinolones and TMP–SMX, with frequent MDR involving fluoroquinolone and TMP–SMX co-resistance.^39^ Similarly, a cross-sectional study on a consecutive sampling of 1262 urine cultures from Houston primary care clinics revealed that 187 (25%) of *E. coli* isolates displayed MDR, with a high resistance to TMP–SMX (44%) and ciprofloxacin (30%), while nitrofurantoin and fosfomycin remained susceptible.^40^ In line with these findings, using a meta-analysis of a consecutive sampling total of 1893 UPEC isolates identified ESBL production among 392 UPEC isolates, especially CTX-M-1, as the most prevalent resistance mechanism in UPECs in Mexico.^41^ As per a retrospective observational US cohort study, consecutive sampling of 12 234 recurrent uUTIs patients in the USA had substantially higher odds of MDR and increased resistance to TMP–SMX (21.8%) and fluoroquinolones (14.2%) compared with those with 68 033 non-recurrent uUTIs.^41^ Paediatric populations in the USA also exhibit a heightened prevalence of ESBL and MDR phenotypes, as per a multi-centre, national surveillance-based study utilizing data from the SENTRY Antimicrobial Surveillance Program across 89 medical centres in the USA, particularly in infants and young children (0–24 months), with the highest resistance rates of 10.8% ESBL resistance from the analysis of 3511 *E. coli* isolates from paediatric patients with UTI observed in the Pacific region.^42^ Nitrofurantoin and fosfomycin demonstrate low resistance rates, suggesting their continued efficacy for treating uncomplicated UTIs.^43^

### South America

In South America, antimicrobial resistance among UPEC isolates differed across countries and patient populations, with 768 UPEC isolates from elderly hospitalized patients of over 70 years old with UTIs without urinary catheters and no antimicrobial therapy in a single-centre, hospital-based cross-sectional study in Argentina exhibiting a notably higher rate of about 80.5% ampicillin-resistant isolates.^44^ However, in these patients, resistance to third-generation cephalosporins was ˂10% and carbapenem resistance was not detected. However, in northeastern Brazil, resistance to ciprofloxacin was 24.4% and sulfamethoxazole-trimethoprim 50.6%, although resistance to third-generation cephalosporins remained lower than 10%.^45^ A 2017 hospital-based study conducted in Cusco, Peru on a consecutive sampling of 99 ESBL-producing UPEC isolates reported that over 80% were resistant to both ciprofloxacin and TMP–SMX, the two commonly used therapies for UTIs.^46^ The UPEC isolates from Peru exhibited resistance to aminoglycosides, nitrofurantoin and cefoxitin, reflecting similar patterns observed in Argentina’s elderly populations. The widespread ciprofloxacin resistance across Brazil, Peru and Argentina indicates a concerning regional trend of MDR, highlighting the need for enhanced antimicrobial stewardship and surveillance programmes.

## Mechanisms of resistance

**Figure 1.**
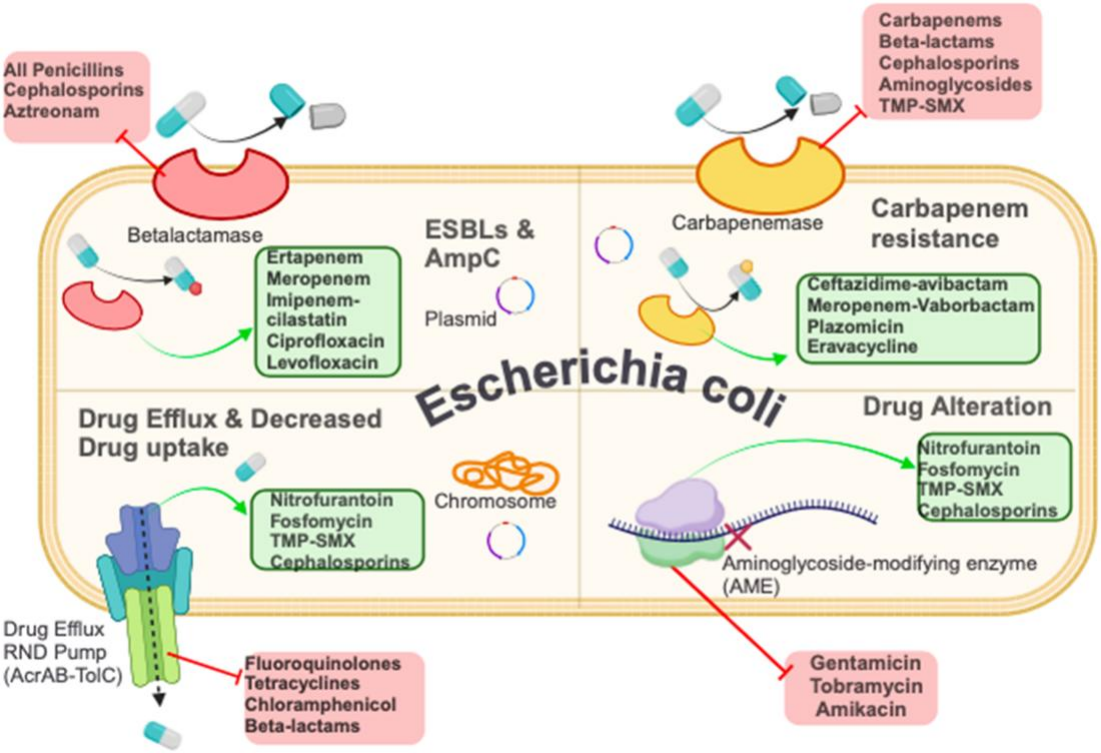
Mechanisms of Resistance in uropathogenic *E. coli* (UPEC) (Image created using BioRender.com).

In Figure 1, the first quadrant is β-lactamase-mediated resistance (ESBLs and AmpC), where uropathogenic *E. coli* (UPEC) produces β-lactamase enzymes (ESBLs and AmpC) that inactivate β-lactam antibiotics, conferring resistance to penicillins and cephalosporins. Effective treatments include ciprofloxacin and ertapenem. Aminoglycosides and TMP–SMX can be used, but with caution, as resistance and toxicity associated with these antibiotics are high. The second quadrant is carbapenemase-mediated resistance, where UPEC expresses carbapenemase enzymes that degrade carbapenems and other β-lactam antibiotics, leading to resistance to cephalosporins and TMP–SMX. Active agents against UPEC include plazomicin and meropenem–vaborbactam. The third quadrant is drug efflux and reduced uptake, where UPEC utilizes efflux pumps to expel antibiotics and reduces membrane permeability to limit drug entry. This mechanism provides resistance to fluoroquinolones and tetracyclines, while nitrofurantoin and TMP–SMX remain effective. The fourth quadrant is drug modification in which aminoglycoside-modifying enzymes (AMEs) alter the structure of aminoglycosides, leading to resistance to gentamicin and tobramycin. Effective alternatives include fosfomycin and cephalosporins.

### ESBL and AmpC-producing *E. coli* and their therapeutic options

ESBL-producing *E. coli* hydrolyse β-lactam antibiotics, including penicillins, cephalosporins and aztreonam. Clinically relevant ESBL-producing *E. coli* belong to class A Ambler classification; SHV, TEM and CTX-M ESBLs are the most common traits among clinical isolates.^2^ AmpC β-lactamase-producing *E. coli* belong to class C of Ambler classification that can be either chromosomally medicated and non-inducible or plasmid mediated.^47^

uUTIs caused by ESBL-producing and AmpC *β-lactamase-*producing *E. coli* pose a significant therapeutic challenge in terms of therapeutic options.^48^ However, oral and intravenous (IV) effective options are available for treating these infections.^49,50^ Nitrofurantoin is a first-line oral agent with excellent urinary concentration for uncomplicated UTIs caused by ESBL and AmpC-producing *E. coli.*^51^ However, it is ineffective in pyelonephritis or systemic infections because it does not achieve therapeutic levels in the bloodstream or kidneys. Nitrofurantoin is contraindicated in patients with severe renal impairment (eGFR <30 mL/min) due to reduced urinary excretion and potential toxicity. TMP–SMX is another oral option for uncomplicated UTIs caused by ESBL and AmpC-producing *E. coli*. It remains a viable option when susceptibility is confirmed via susceptibility testing. Resistance rates have increased and therefore, TMP–SMX is generally avoided in cases of high local resistance (>20%) mediated by mutation in *FolA* or *dfr* genes and in patients with sulpha allergies.^52^ Instances where Nitrofurantoin and TMP–SMX cannot be used, fluoroquinolones, including ciprofloxacin and levofloxacin, can be viable options. However, resistance to fluoroquinolones among ESBL and AmpC-producing *E. coli* has risen sharply, limiting their use. If the organism is susceptible, these agents can be effective due to their high urinary concentration and good oral bioavailability. Fluoroquinolones should be used cautiously due to their risk of collateral damage, including *Clostridioides difficile* infection and tendon rupture.^53^ Fosfomycin is an excellent oral option for treating ESBL and AmpC-producing *E. coli* in uncomplicated UTIs and has a unique mechanism of action, disrupting bacterial cell wall synthesis. Fosfomycin is given as a single-dose oral therapy, which makes it a convenient option, but its efficacy is slightly lower than other agents in complicated cases.^54^ Temocillin is a β-lactam antibiotic resistant to β-lactamase hydrolysis, making it effective against ESBL and AmpC-producing organisms. It has strong urinary excretion and has been used successfully in MDR UTIs. Due to its narrow spectrum of activity, this is often used in cUTIs rather than uUTIs. The ASTARTÉ clinical trial is currently underway to assess whether temocillin is non-inferior to carbapenems.^55,56^ However, availability is limited in some regions, and it is primarily used in Europe.

cUTIs with a variety of underlying comorbidities becomes problematic when caused by MDR *E. coli*. These patients most often end up in the hospital settings and require broad-spectrum and effective antibiotic therapy. The choice of treatment depends on antibiotic susceptibility, infection severity and patient factors.^57^ TMP–SMX was once a first-line UTI treatment, but resistance in ESBL-producing *E. coli* limits its use. It is effective only if susceptibility is confirmed, but resistance rates exceed 30%–50% in many regions. It is a potential alternative in mild-to-moderate cUTIs, particularly in outpatient settings.^58^ Cephalosporins, particularly third- and fourth-generation cephalosporins, are commonly used for Gram-negative cUTIs. These include cefepime, ceftazidime, ceftriaxone and cefotaxime but with variable efficacy. Cephamycins, such as cefoxitin and cefotetan, have intrinsic stability against ESBLs but generally hydrolysed by AmpC β-lactamases. They are primarily used in mild-to-moderate cUTIs, but not for severe infections or urosepsis.^59^ Temocillin is a narrow-spectrum β-lactam resistant to ESBL and AmpC hydrolysis but not ideal in pyelonephritis or bacteraemia.^55,56^ Although, fosfomycin is mainly used as a single-dose treatment for uncomplicated UTIs, IV fosfomycin is an option for cUTIs, particularly for biofilm-associated infections. However, its use in systemic infections is still under investigation, and resistance can develop with monotherapy.^54^ Fluoroquinolones such as ciprofloxacin and levofloxacin are alternatives only if susceptibility is confirmed and in patients without prior fluoroquinolone exposure. Furthermore, severe side effects (e.g. tendon rupture, QT prolongation and neurotoxicity), has discouraged clinicians using fluoroquinolones in these circumstances.^7^ Aminoglycosides such as gentamicin, amikacin and tobramycin are potent bactericidal agents used in synergy with β-lactams for severe cUTIs. Amikacin is preferred over gentamicin due to higher stability against AMEs found in MDR bacteria. They have excellent activity against ESBL and AmpC-producing *E. coli,* but nephrotoxicity and ototoxicity limit long-term use. These antibiotics are reserved for severe infections, often as once-daily dosing (concentration-dependent killing).^60^ Carbapenems such as meropenem and imipenem are highly effective in cUTIs and generally need IV administration. Ertapenem may be used as a once-daily IV treatment in outpatient settings. Doripenem is a broad-spectrum carbapenem with strong activity against ESBL and AmpC-producing *E. coli* in cUTIs and it has lower seizure risk than imipenem and better activity than meropenem. Reserved for critically ill patients, particularly those with urosepsis, bacteraemia or severe pyelonephritis.^61^ β-Lactam/β-Lactamase Inhibitor (BL-BLI) Combinations inhibits β-lactamase enzymes produced by MDR bacteria, restoring the activity of β-lactam antibiotics.^62,63^ Piperacillin–tazobactam is effective against ESBL-producing *E. coli*, but its efficacy in high-inoculum infections (e.g. severe pyelonephritis and urosepsis) is debated, as it may be less effective against AmpC producers. Therefore, it is moderately used in cUTIs when susceptibility is confirmed.^64^ ceftazidime–avibactam can be used against ESBL, AmpC and some CREC, but ineffective against metallo-β-lactamases (MBLs). Meropenem–vaborbactam and imipenem/cilastatin/relebactam combinations are reserved for carbapenem-resistant infections but may be overkill for non-carbapenemase-producing ESBL infections.^65^

### Carbapenem-resistant *E. coli* and their therapeutic options

CREC harbour carbapenemase and metallo-beta-lactamases encoded by *blaKPC, blaNDM, blaOXA-48, blaOXA-232, blaOXA-181, blaIMP* or *blaVIM* genes.^66^ These strains pose a severe therapeutic challenge as they inactivate carbapenems, often the last line of defence for Gram-negative infections. In addition to carbapenem resistance, they also harbour genes that offer resistance to a number of other antibiotics such as β-lactam, fluoroquinolone, aminoglycosides, sulphonamides, tetracyclines, macrolides, fosfomycin and others, rendering them super bugs. Some strains of CREC may be susceptible to nitrofurantoin and TMP–SMX that can be used, but most often in uncomplicated UTIs. Ciprofloxacin and levofloxacin are fluoroquinolones that work by inhibiting bacterial DNA gyrase and topoisomerase IV, preventing DNA replication, are useful if susceptible as the risk of resistance in CREC is high. Ciprofloxacin is usually prescribed orally every 12 h, while levofloxacin is given once daily.^67,68^ Aminoglycosides such as gentamicin, amikacin and tobramycin work by binding to bacterial ribosomes, disrupting protein synthesis and are effective against susceptible CREC infections, given as a single-dose treatment. Aminoglycosides are often used in combination with other antibiotics for severe systemic infections for a synergistic effect. Dosing requires careful monitoring of drug levels to minimize toxicity while ensuring efficacy.^60,69^ Fosfomycin is an oral antibiotic used to treat uncomplicated UTIs caused by *E. coli*, including some CREC strains. While effective for cystitis, fosfomycin is not recommended for pyelonephritis or systemic infections due to its low serum levels. It is generally well tolerated, with mild gastrointestinal side effects being the most common.^54^ Colistin and polymyxin B are last-resort antibiotics used for treating severe CREC complicated UTIs. They disrupt bacterial cell membranes, leading to cell death. Due to their high nephrotoxicity and neurotoxicity, these drugs are typically reserved for MDR infections when other options are unavailable. Colistin is usually given as a loading dose followed by maintenance dosing, adjusted based on renal function. Polymyxin B is preferred in cases of renal impairment since it does not require dose adjustments based on kidney function.^70^ BL-BLI combinations such as ceftazidime–avibactam can be given IV every 8 h. It has demonstrated good efficacy with a more favourable safety profile than colistin-based regimens. However, some CREC strains, particularly those producing MBLs, remain resistant to ceftazidime–avibactam.^71^ Meropenem–vaborbactam, a combination of the carbapenem meropenem and the β-lactamase inhibitor vaborbactam, is used for complicated UTIs and the usually given IV every 8 h. It has shown superior efficacy and lower toxicity compared with colistin-based treatments. However, it is ineffective against CREC strains that produce MBLs (e.g. NDM-1).^72^ Imipenem–cilastatin–relebactam is another BL-BLI combination that provides enhanced activity, where Imipenem is a carbapenem, cilastatin prevents its renal degradation and relebactam extends its spectrum by inhibiting β-lactamases. This combination is used for complicated UTIs and CRECs, and given IV every 6 h. It has a favourable safety profile compared with older polymyxin-based therapies.^65^ Cefiderocol is a novel siderophore cephalosporin that exploits bacterial iron transport systems to gain entry into cells, making it highly effective against MDR Gram-negative pathogens, including CREC. Cefiderocol is particularly useful against MBL-producing CREC strains, which are resistant to many other agents. Its tolerability profile is similar to that of other cephalosporins, with gastrointestinal symptoms and infusion-related reactions being the most common side effects.^73^ Combination therapies of two different classes of antibiotics for synergistic activity are often used to enhance bacterial eradication, prevent resistance development and improve clinical outcomes. Given the high mortality associated with CREC infections, a β-lactam agent is frequently combined with other antibiotic classes such as aminoglycosides (gentamicin, amikacin and tobramycin), fluoroquinolones (ciprofloxacin and levofloxacin), tetracyclines (eravacycline and tigecycline), colistin or polymyxins to provide synergistic activity against these highly resistant bacteria.

### Non-β-lactam resistance and their therapeutic options

Aminoglycoside resistance arises due to AMEs, ribosomal mutations and efflux pumps, diminishing the effectiveness of drugs like gentamicin and amikacin. Fosfomycin resistance is mediated by enzyme-mediated degradation (fosA gene), mutations in transport proteins and target site modifications, restricting its use in MDR cUTIs. Additionally, sulphonamide and trimethoprim resistance are caused by mutations in the *dfr* and *sul* genes, which alter dihydropteroate synthase and dihydrofolate reductase, rendering these antibiotics ineffective. The widespread emergence of these resistance mechanisms significantly limits therapeutic options for cUTIs, necessitating alternative treatment strategies, novel antimicrobial agents and strict infection control measures to curb the spread of MDR pathogens.^74^ Table 1 summarizes the resistance mechanisms in cUTIs.

**Table 1.** Summarizing the resistance mechanisms in complicated UTIs

Resistance mechanism	Key resistance features	Common therapeutic options
ESBLs producers (uncomplicated and complicated)	Hydrolyse penicillins, cephalosporins and aztreonam but not carbapenems	Requires carbapenems or β-lactam/β-lactamase inhibitors
AmpC β-lactamase producers	Resistance to cephalosporins and monobactams, inducible expression	Carbapenems are preferred; avoid cephalosporins
CREC	Enzymatic degradation (KPC, NDM, VIM and OXA-48), efflux pumps and porin loss	Requires polymyxins, tigecycline or novel β-lactam/β-lactamase inhibitors
Fluoroquinolone resistance	Mutations in gyrA, parC (topoisomerases) and efflux pumps	Requires alternative agents (e.g. β-lactams and aminoglycosides)
Aminoglycoside resistance	AMEs, 16S rRNA methylation and efflux pumps	Limited use; often combined with β-lactams for synergism
Fosfomycin resistance	FosA enzyme, mutations in transporters (GlpT and UhpT)	Limited oral and IV options

## Emerging antibiotics against MDR Gram-negative bacteria

The rise of MDR Gram-negative bacteria has led to the development of novel antibiotics and β-lactam/β-lactamase inhibitor combinations.^6,43,75–77^ Table 2 lists some emerging antibiotics to combat CREC and other Gram-negative bacteria including *Enterobacterales*, *P. aeruginosa* and *Acinetobacter baumannii*. Table 2 summarizes some of the newer combinations that are being evaluated in clinical trials for cUTIs caused by MDR pathogens including *E. coli*.

**Table 2.** Novel antibiotics for MDR Gram-negative bacteria

Novel antibiotic	Category	Activity	Indications	Advantages
Sulopenem^78^	Novel penem-class β-lactam antibiotic	ESBL-producers and carbapenem-resistant strains	cUTIs, intra-abdominal infections and respiratory infections	Lower risk of resistance Oral formulation will allow outpatient therapy
Tebipenem^79^	Novel carbapenem	Mainly ESBL producers	Ear, nose, throat, respiratory infections and cUTIs	Good oral option for cUTIs
Taniborbactam + Cefepime^61^	Taniborbactam (next-generation β-lactamase inhibitor) and cefepime (fourth-generation cephalosporin)	Wide range of β-lactamases, including, KPC, OXA and MBL like NDM	cUTIs and hospital-acquired infections	Coverage against difficult to treat MBL producers, which are typically resistant to most β-lactams
Enmetazobactam + Cefepime^80^	Enmetazobactam (ESBL inhibitor) and cefepime (fourth-generation cephalosporin)	ESBL-producers and carbapenem-resistant strains	cUTIs and bloodstream infections caused by MDR Enterobacterales	Higher efficacy than piperacillin–tazobactam and promising alternative to carbapenems
Zidebactam + Cefepime^81^	Zidebactam (a unique β-lactam enhancer + cefepime (fourth-generation cephalosporin)	carbapenem-resistant strains	cUTIs, pneumonia and bloodstream infections	Reduces the need for polymyxins or aminoglycosides
Nacubactam + Meropenem^82^	Nacubactam (novel diazabicyclooctane [DBO]—β-lactamase inhibitor) and meropenem (carbapenem)	KPC-producing, OXA-producing and some MBL-producing Enterobacterales	cUTIs, pneumonia and complicated intra-abdominal infections	Also inhibits PBP2, making it more effective against carbapenem-resistant pathogens

## Duration and oral transition in complicated UTIs

The optimal duration of antimicrobial therapy for complicated urinary tract infections (cUTIs) and urosepsis has undergone a major paradigm shift in recent years. Historically, treatment regimens commonly extended for 10–14 days or longer, driven by concerns regarding relapse, persistent bacteraemia and complications in vulnerable patient populations. However, accumulating evidence from randomized controlled trials, meta-analyses and updated international guidelines now supports shorter, individualized treatment courses, provided that effective source control is achieved (e.g. relief of obstruction, catheter removal or exchange) and that the patient demonstrates clinical improvement (Table 3). For patients with cUTI without associated bacteraemia, a 5–7-day course of effective antimicrobial therapy is typically sufficient, particularly when source control is prompt. The European Association of Urology (EAU) recommends 7–10 days for complicated cases, with shorter durations appropriate for catheter-associated infections when managed properly.^83^ In cases of Gram-negative bacteraemia of urinary origin, landmark randomized controlled trials.^85^ have shown that 7 days of therapy is non-inferior to 14 days regarding mortality, relapse and readmission when patients are clinically stable and have no uncontrolled source. Consequently, both the EAU and the Infectious Diseases Society of America now endorse 7 days as the standard duration for most cases of bacteraemic cUTI once stabilization occurs.^84^

**Table 3.** Therapeutic duration and key considerations

Clinical scenario	Recommended duration	Key considerations	Preferred oral agents	References
cUTI without bacteraemia	5–7 days	Shorter course if source control is achieved (e.g. catheter removal, obstruction relieved). Longer (7–10 days) if delayed response or severe illness	Fluoroquinolone, TMP–SMX, oral β-lactams (if susceptible)	Kranz et al^83^; Nicolle et al^84^
CAUTI	5–7 days	Catheter should be removed or replaced; shorter durations effective when clinical response is prompt	Fluoroquinolone, TMP–SMX, oral β-lactams	Kranz et al^83^; Nicolle et al^84^
cUTI with Gram-negative bacteraemia (clinically stable)	7 days	Supported by RCTs showing non-inferiority of 7 versus 14 days; requires effective source control and stable clinical status	Fluoroquinolone, TMP–SMX, oral β-lactams, fosfomycin (selected cases)	Tamma et al^6^; Yahav et al^85^
cUTI with bacteraemia and delayed clinical response	10–14 days	Extend duration if persistent fever/bacteraemia, immunocompromised status, or inadequate source control	Based on susceptibility and site penetration	Kranz et al^83^; Nicolle et al^84^
Prostatitis/prostatic involvement	2–4 weeks	Longer courses required for adequate prostatic penetration; choice of antibiotic should consider tissue distribution	Fluoroquinolones (good prostatic penetration), TMP–SMX	Kranz et al^83^; Nicolle et al^84^
Immunocompromised or structural abnormality (e.g. transplant)	10–14 + days	Tailor based on clinical response and comorbidities; often require closer follow-up and sometimes prolonged therapy	Tailored to susceptibility; often fluoroquinolones or TMP–SMX	Kranz et al^83^; Nicolle et al^84^

Importantly, recent evidence has also strengthened the role of oral step-down therapy in managing cUTIs, including those presenting with uroIDS. Traditionally, prolonged IV courses were the norm; however, recent studies indicate that in clinically stable patients with adequate source control and pathogen susceptibility, early transition from IV to oral therapy—typically, after 48 to 72 h of effective IV treatment—are both safe and effective. Transition is appropriate once the patient achieves hemodynamic stability, remains afebrile for 24–48 h, shows improving laboratory parameters, and can tolerate oral intake, provided an active oral agent with suitable bioavailability is available. Evidence suggests that total treatment duration of ∼7 days (IV + oral combined) is sufficient for most stable cases of bacteraemic cUTI. Meta-analyses^86^ have confirmed these findings, showing no increase in treatment failure or mortality with shorter courses and early oral switch. For non-bacteraemic or catheter-associated infections, 5–7 days of total therapy is generally adequate when source control is achieved. Longer courses (10–14 days or more) or durations based on patient assessment are indicated for patients with delayed clinical responses, persistent bacteraemia, immunocompromised states, structural abnormalities, prostatitis or infections caused by organisms without suitable oral options. In men with suspected or confirmed prostatitis, therapy often extends to 2–4 weeks, depending on pathogen and response. The choice of oral agent is crucial: fluoroquinolones and TMP–SMX are the most validated due to excellent bioavailability and urinary penetration, though appropriately dosed oral β-lactams and fosfomycin have also shown promise in selected cases. By adopting structured IV→oral transition strategies, clinicians can achieve effective treatment outcomes while minimizing unnecessary IV exposure, reducing hospital stay, lowering drug-related adverse events, improving patient comfort and supporting antimicrobial stewardship.

## Alternative therapeutic agents

With the rise of antibiotic resistance and recurrent UTIs, alternative therapeutic strategies have gained increasing attention. These approaches focus on prevention and treatment through non-antibiotic means, targeting host immunity, bacterial virulence and bladder health. Below are key alternative therapies categorized into preventive and treatment approaches.

### Topical oestrogen therapy

Recurrent UTIs are common in post-menopausal women, largely due to oestrogen deficiency. Oestrogen plays a critical role in maintaining urogenital health by supporting vaginal microbiota, epithelial integrity and immune defences. One of the alternative therapeutic approaches for preventing UTIs in post-menopausal women is the topical oestrogen therapy. Oestrogen deficiency leads to thinning of the vaginal and urethral epithelium, loss of glycogen and a decline in *Lactobacillus* species. This increases the risk of uropathogens colonization (e.g. *E. coli*). Oestrogen therapy restores the urothelial barrier, promotes *Lactobacillus* growth, and lowers vaginal pH (maintaining an acidic environment that inhibits pathogen growth). It also increases AMP production and strengthens mucosal immunity.^87^

The different forms of topical oestrogen therapy are (i) vaginal oestrogen creams that are applied directly inside the vagina. It improves vaginal tissue thickness and pH balance. Examples: oestradiol (0.01%) and conjugated oestrogen creams (ii) vaginal oestrogen rings or estring that is a flexible silicone ring placed inside the vagina, releasing oestradiol continuously for 3 months. It maintains consistent oestrogen levels with minimal systemic absorption. (iii) Vaginal oestrogen tablets or oestradiol tablets (vagifem and imvexxy) placed inside the vagina, which are effective in restoring vaginal epithelium and preventing UTIs. The clinical evidence and effectiveness of the studies show that topical oestrogen therapy reduces UTI recurrence by up to 50% in post-menopausal women. Unlike systemic oestrogen therapy (e.g. oral oestrogen), vaginal oestrogen has minimal systemic absorption, reducing risks associated with hormone therapy.^88^

### Probiotics

Probiotics, particularly *Lactobacillus* species, play a crucial role in preventing UTIs by maintaining a healthy vaginal and gut microbiome. *Lactobacillus crispatus*, *Lactobacillus rhamnosus* GG and *Lactobacillus reuteri* produce lactic acid, which lowers vaginal pH, creating an environment that is hostile to uropathogens such as *E. coli*. Additionally, these beneficial bacteria generate hydrogen peroxide (H_2_O_2_) with antimicrobial properties and compete for adhesion sites on the uroepithelial surface, preventing pathogen colonization. Probiotics can be taken orally in capsule form or administered intravaginally as suppositories. Clinical studies suggest that regular probiotic use can reduce UTI recurrence by 40%–50%, especially when combined with other preventive measures like cranberry extract or D-mannose.^89,90^

### Cranberries

Cranberries contain bioactive compounds called proanthocyanidins (PACs), which prevent uropathogenic *E. coli* from adhering to the bladder wall, thereby reducing bacterial colonization and the likelihood of infection. Unlike antibiotics, cranberries do not kill bacteria but instead interfere with bacterial adhesion, minimizing the risk of antibiotic resistance. Cranberries are available in various forms, including unsweetened juice, capsules and tablets, with cranberry extract supplements offering a more concentrated and effective dose of PACs. Studies indicate that consuming at least 36 mg of PACs per day can lower the risk of recurrent UTIs by up to 30%. However, cranberry juice often contains high amounts of sugar, making capsules or tablets a preferred option for those seeking effective UTI prevention.^91,92^

### D-mannose

D-Mannose is a naturally occurring sugar that acts as a powerful bacterial antiadhesive, specifically targeting uropathogenic *E. coli*, the most common cause of UTIs. When consumed, D-mannose binds to bacterial fimbriae, preventing the bacteria from attaching to the bladder lining and allowing them to be easily flushed out with urine. It is available in powder or capsule form, with recommended doses of 2 g/day for prevention and 3 g/day for acute UTI episodes. Clinical trials have demonstrated that D-mannose is as effective as nitrofurantoin in preventing recurrent UTIs, with a 45%–50% reduction in UTI recurrence. Additionally, because it does not affect beneficial gut microbiota, it is a safe alternative to antibiotics with minimal side effects.^93–95^

### Methenamine hippurate

Methenamine hippurate has been widely used as a preventive measure in countries such as those in Scandinavia, although its efficacy compared with antibiotics remains debatable. In the urine, methenamine is converted into formaldehyde, which lowers the pH, creating an acidic environment that is hostile to bacteria and helps sterilize the urine.^96,97^ While several systematic reviews have reported no significant benefit of methenamine hippurate over antibiotics, recent clinical trials suggest that it is an effective prophylactic agent, capable of reducing recurrent UTIs by up to 25% compared with low-dose antibiotic regimens.^98^

### Glycosaminoglycan layer substituents

The bladder's glycosaminoglycan (GAG) layer serves as a protective barrier that prevents bacteria and toxins from adhering to and penetrating the bladder epithelium. Chronic or recurrent UTIs can lead to GAG layer damage, making the bladder more susceptible to infections and inflammation. Restoring this layer with intravesical instillation of hyaluronic acid and chondroitin sulphate has been shown to reduce UTI recurrence and improve bladder health, particularly in individuals with interstitial cystitis or recurrent bacterial cystitis. Unlike oral supplements, GAG therapy is administered directly into the bladder via catheterization and is most beneficial for patients with chronic UTI-related bladder damage. Studies support its effectiveness in reducing bacterial adhesion and inflammation, offering a promising alternative therapy for those experiencing frequent infections.^99^

### Nanoparticles

Nanoparticles represent a novel and promising approach in the treatment of UTIs, particularly for combating antibiotic-resistant bacteria. These microscopic particles often made from materials such as silver, gold, chitosan or lipid-based carriers, exhibit potent antimicrobial properties by directly interacting with bacterial cell membranes, leading to bacterial destruction. Silver nanoparticles, for example, generate reactive oxygen species that damage bacterial DNA and proteins, while lipid-based nanoparticles can enhance drug delivery by increasing the bioavailability of antibiotics at the infection site. Studies suggest that nanoparticle-based treatments can effectively eliminate uropathogenic *E. coli* and other resistant strains while minimizing toxicity and reducing the risk of antibiotic resistance. This emerging technology holds great potential for enhancing conventional antibiotic therapies and providing alternative treatment options for persistent and MDR UTIs.^47,100,101^

### Bacteriophages

Bacteriophages, or phages, are viruses that selectively infect and lyse bacterial cells, offering a highly targeted therapy for UTIs caused by antibiotic-resistant pathogens. Unlike broad-spectrum antibiotics, which can disrupt the gut and vaginal microbiota, phages specifically attack uropathogenic bacteria such as *E. coli, Klebsiella pneumoniae* and *P. aeruginosa*, leaving beneficial bacteria unharmed. Phage therapy is particularly useful for patients with recurrent UTIs who no longer respond to conventional antibiotics. Some studies have demonstrated the effectiveness of phage cocktails in eradicating biofilms—protective layers that bacteria form to evade antibiotics—making phages a powerful alternative for chronic and complicated UTIs. While still under clinical investigation, bacteriophage therapy represents a promising, personalized approach for treating drug-resistant infections.^102,103^

### Predatory bacteria

Predatory bacteria, such as *Bdellovibrio bacteriovorus* and *Micavibrio aeruginosavorus*, are naturally occurring microbes that hunt and consume pathogenic bacteria, including UTI-causing species. These unique bacteria penetrate the outer membrane of Gram-negative pathogens like *E. coli* and *K. pneumoniae*, rapidly replicating within the host and ultimately destroying it. Unlike conventional antibiotics, predatory bacteria selectively target harmful microbes without disturbing the natural microbiota, reducing the risk of dysbiosis and antibiotic resistance. Early research suggests that these self-replicating ‘living antibiotics’ could be a viable option for managing MDR UTIs, although further studies are needed to assess their safety and efficacy in clinical settings.^104,105^

### Antimicrobial peptides

AMPs are naturally occurring molecules found in the immune systems of humans, animals and plants, with potent antibacterial properties. These peptides work by disrupting bacterial membranes, inhibiting biofilm formation and modulating the immune response to enhance bacterial clearance. Examples include cathelicidins, defensins and synthetic peptides designed to target specific UTI pathogens. Unlike traditional antibiotics, AMPs exhibit a lower tendency to induce resistance because of their rapid and multi-target mechanism of action. Some AMPs, such as LL-37 and melittin, have demonstrated strong activity against uropathogens, including antibiotic-resistant *E. coli* and *K. pneumoniae*. Ongoing research aims to optimize AMPs for therapeutic use, potentially leading to new treatment options for recurrent and antibiotic-resistant UTIs.^106–108^

### UTI vaccines

Given the complications in managing MDR *E. coli* in UTIs, vaccines represent a viable approach for preventing and treating these infections. Several vaccines for UTIs are currently in clinical trials, and a few have been approved for use in certain countries. However, selecting an appropriate vaccine candidate is challenging. Humoral immunity may only prevent bacterial adhesion to uroepithelial cells and may not be effective against established infections. In contrast, T cells can recognize and eliminate established pathogens. Therefore, strategies that address both the prevention and eradication of established UTIs caused by MDR *E. coli* are of prime importance. Whole-cell inactivated vaccines such as Uromune, Uro-Vaxom and Solco-Urovac have entered the market. In addition, live attenuated vaccines, as well as subunit and conjugate vaccines, are currently under investigation.^109^

## Conclusion

The management of cUTIs and urosepsis caused by MDR pathogens remains a significant clinical challenge due to the rising prevalence of MDR *E. coli*, including ESBL and ampC-producing, and CREC strains. While conventional antibiotics are often ineffective, novel BL-BLI combinations such as ceftazidime–avibactam, meropenem–vaborbactam, imipenem–cilastatin–relebactam and cefiderocol have shown improved efficacy against MDR *E. coli*, in order to sparingly use the last-resort drugs like carbapenems and polymixins. Additionally, alternative and adjunct therapies, including nanoparticles, bacteriophages, predatory bacteria and AMPs, offer promising avenues for treatment, particularly for antibiotic-resistant infections. Non-antibiotic preventive strategies, such as probiotics, D-mannose, cranberry extracts and GAG layer therapy, play a crucial role in reducing recurrent infections, thereby minimizing antibiotic use and resistance development. Moving forward, a multi-disciplinary approach combining novel antimicrobials, innovative therapies and infection control measures will be essential in effectively managing MDR *E. coli* in uUTIs and cUTIs mitigating the global threat of antibiotic resistance.
