# Correction to: Therapeutic strategies for multi-resistant *Escherichia coli* in urinary tract infections—a cause for concern

**DOI:** 10.1093/jacamr/dlag107

**Published:** 2026-06-04

This is a correction to: Rekha Unni, Ruby Varghese, Benita Arakal, Lochana Padmanabhan, Srijita Karmakar, Achinthya Sampathila, Yogesh Bharat Dalvi, Paul Livingstone, Therapeutic strategies for multi-resistant *Escherichia coli* in urinary tract infections—a cause for concern, *JAC-Antimicrobial Resistance*, Volume 8, Issue 3, June 2026, dlag081, https://doi.org/10.1093/jacamr/dlag081

In the originally published version of this manuscript, there was a typographical error in the name of the fifth-listed author.

This has been corrected.

